# The Use of Ossein–Hydroxyapatite Complex in Conjunction with the Ilizarov Method in the Treatment of Tibial Nonunion

**DOI:** 10.3390/jcm14103353

**Published:** 2025-05-12

**Authors:** Piotr Morasiewicz, Monika Zaborska, Michał Sobczak, Łukasz Tomczyk, Daniele Pili, Krystian Kazubski, Paweł Leyko

**Affiliations:** 1 Department of Orthopaedic and Trauma Surgery, Institute of Medical Sciences, University of Opole, Witosa 26, 45-401 Opole, Poland; 2 Institute of Medical Sciences, Faculty of Medicine, University of Opole, Witosa 26, 45-401 Opole, Poland; 3 Department of Food Quality and Safety Management, Faculty of Food Science and Nutrition, Poznan University of Life Sciences, Wojska Polskiego 28, 60-637 Poznan, Poland; 4 Orthopedic and Trauma Department, G B. Mangioni Hospital, Via L. Da Vinci 49, 23900 Lecco, Italy

**Keywords:** ossein–hydroxyapatite complex, osteogenon, Ilizarov method, tibial nonunion, bone nonunion

## Abstract

**Background:** Patients with nonunion experience pain, mobility problems, and physical activity limitations; require long-term, costly treatment; and cannot resume work. Some authors recommend the use of pharmaceutical agents as an adjunct therapy in fracture and nonunion treatment. The aim of this study was to assess the effects of ossein–hydroxyapatite complex used as an adjunct therapy in nonunion treatment with the Ilizarov external fixator. **Methods:** In this retrospective study, we assessed 31 patients (nine women, 22 men) at a mean age of 47 years (29–68 years), who were receiving osteogenon, with aseptic tibial shaft nonunion treated with the Ilizarov external fixator in the period 2019–2023, designated as Group 1. The control group comprised 29 patients (five women, 24 men), at a mean age of 48 years, with aseptic tibial shaft nonunion treated with the Ilizarov external fixator, who did not receive osteogenon during treatment, designated as Group 2. We assessed the following parameters—duration of Ilizarov fixation, achieved bone union, time to resuming normal physical activity, maintained bone union, time to complete pain relief, the number of patients reporting complete pain relief, the number of patients who were fitted with a cast or splint following Ilizarov fixator removal, and the rate of complications. **Results:** The median time to Ilizarov fixator removal was 275 days in Group 1 and 218 days in Group 2. In Group 1, bone union was observed in 100% of patients, in Group 2, 93% of patients achieved bone union. This difference was statistically significant, *p* = 0.041. Maintained bone union was observed in 85.7% of patients from the osteogenon group and in 79.3% of patients from the control group, and the difference was not statistically significant. There were no differences between groups in the median time to resuming normal physical activity, the median time to achieving pain relief, the rate of complications, and the rate of pain relief. **Conclusions:** The use of ossein–hydroxyapatite complex has a beneficial effect on fracture nonunion treatment with the Ilizarov method. The use of osteogenon helps increase the proportion of patients with fracture nonunion who achieve bone union following treatment with the Ilizarov method. Osteogenon does not significantly affect complication rates, time to fixator removal, time to achieving pain relief, time to resuming normal physical activity, maintained bone union rates, or the proportion of patients who achieve pain relief.

## 1. Introduction

Problems with fracture healing, delayed union, and nonunion continue to pose considerable orthopedic challenges. According to epidemiological data, nonunion occurs in approximately 4–10% of all fractures [1,2,3]. In most cases, nonunion occurs in long bones, particularly in the tibia (3–14% of tibial fractures) [1,2,3,4]. Patients with nonunion experience pain, mobility problems, and physical activity limitations; require long-term, costly treatment; and cannot resume work [1,3,4]. Therefore, surgical treatment is indicated in cases of nonunion. The Ilizarov method is an effective treatment for tibial nonunion that is adopted worldwide [4,5,6,7,8,9,10,11]. The main goals of surgical treatment of nonunion are to achieve strong bone union as soon as possible, to relieve pain, to help the patient return to their normal everyday activities and resume work, and to complete treatment quickly [1,3,4,5,8,10]. Unfortunately, the treatment of tibial nonunion, even conducted with the Ilizarov method, is often associated with prolonged treatment due to a long time required to frame removal, a risk of complications, and persistent pain [4,5,6,7,8,9,10,11]. Nevertheless, bone union cannot be achieved in some cases of tibial fracture treatment with the Ilizarov method, with rates of successful union reported at 96–100% [4,5,6,7,8,9,10,11]. Even after bone union is achieved, some patients with tibial nonunion treated with the Ilizarov method (4–31.6%) may suffer refracture during a long-term follow-up [5,6,7]. Therefore, a novel parameter called “maintained bone union” has been introduced in assessing nonunion treatment at a long-term follow-up [5].

Risk factors for nonunion include osteoporosis and vitamin D deficiency [1]. Some authors recommend the use of pharmaceutical agents as an adjunct therapy in fracture and nonunion treatment [12,13,14,15,16,17,18,19,20,21,22]. Apart from calcium and vitamin D, such agents include osteogenon. Osteogenon is an ossein–hydroxyapatite complex, which stimulates bone remodeling and has been licensed for use as an adjunct therapy in the treatment of fractures and osteoporosis [12,13,14,15,16,17,18,19,20,21,22]. The mechanism of action involves stimulating osteoblasts, inhibiting osteoclasts, supplying structural materials for new bone tissue formation, activating osteogenesis, stimulating bone metabolism, accelerating callus formation, and increasing bone mass [12,13,14,15,16,17,18,19,20,21,22]. Osteogenon contains substances that have a positive effect on osteogenesis and osteoblast proliferation: insulin-like growth factors 1 and 2, collagen type I, and transforming growth factor beta [12,13,14,15,16,17,18,20,22]. The inorganic, mineral component of osteogenon—hydroxyapatite—inhibits bone tissue resorption [12,14,15,17,18,20,22]. This effect of osteogenon may be beneficial in nonunion treatment.

To date, the effects of osteogenon on fracture treatment have not been fully investigated or described [12,13,14]. We were able to find only one study assessing a limited number of parameters following femoral or tibial nonunion treatment with osteogenon in 15 patients [15]. There have been no studies conducted in large patient populations to assess the impact of osteogenon on tibial nonunion treatment with the Ilizarov method.

We posited the hypothesis that the use of osteogenon would have a beneficial effect on fracture nonunion treatment with the Ilizarov method. The purpose of this study was to assess the effects of ossein–hydroxyapatite complex used as an adjunct therapy in nonunion treatment with the Ilizarov method.

## 2. Material and Methods

### 2.1. Study Design

**Figure 1:**
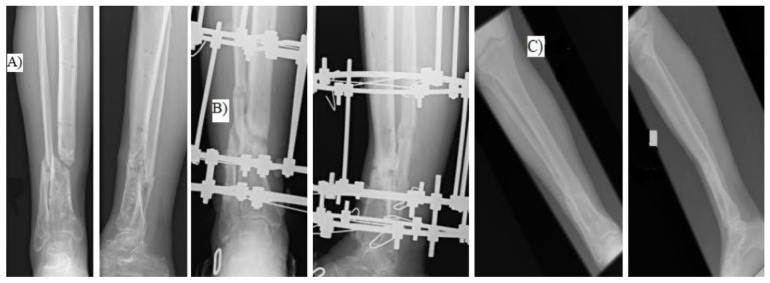
(**A**) patient before surgery, (**B**) patient during treatment with the Ilizarov method, (**C**) long-term result.

We retrospectively assessed patients with aseptic tibial shaft nonunion treated with the Ilizarov external fixator in the period 2019–2023 (Figure 1).

### 2.2. Inclusion and Exclusion Criteria

Study inclusion criteria were tibial shaft nonunion treated with the Ilizarov method, absence of infection, complete medical records, complete radiological records, limb shortening by <1 cm, receiving osteogenon (2 tablets per day, in 12 h intervals) over the entire duration of treatment with the Ilizarov method, receiving no other medications that might affect bone metabolism and remodeling, and a follow-up period of over 2 years after treatment completion. Study exclusion criteria were no treatment with the Ilizarov method, infection, inaccessible medical and radiological records, limb shortening by >1 cm, bone grafting, a follow-up period of <2 years, receiving other medications that may affect bone metabolism. Due to a retrospective nature of this study and the fact that osteogenon had already been approved for use in clinical practice, this study did not require an ethics committee’s approval in accordance with Polish regulations in force at the time the study began.

### 2.3. Participants

Application of the inclusion and exclusion criteria helped select 31 patients (9 women, 22 men) at a mean age of 47 years (29–68 years) who were receiving osteogenon (2 tablets per day) over the entire treatment period (from the mounting to the removal of the Ilizarov external fixator). A single tablet contained beta-transforming growth factor, type I collagen, 178 mg of calcium, insulin-like growth factors 1 and 2, and 82 mg of phosphorus [12,13,14,15,16,17,18,19,20,21]. The control group comprised 29 patients (5 women, 24 men), at a mean age of 48 years, with aseptic tibial shaft nonunion treated with the Ilizarov external fixator, who did not receive osteogenon during treatment. All patients from both groups were operated on by one orthopedic surgeon (the same one). All control group patients had been treated with the Ilizarov external fixator, during an earlier period (2012–2019), when osteogenon had not yet been used as an adjunct therapy.

### 2.4. Procedure

Patients from both groups underwent bone fragment reduction and external fixation of the tibia at the site of nonunion with the Ilizarov method, without bone grafting or bone fragment transport. The operations were performed in the supine position. Under intraoperative X-ray control, the Ilizarov fixator was mounted on the bone fragments. Depending on the size of the bone fragments, each of the bone fragments was stabilized with 1 or 2 rings with K-wires. After stabilization and reposition of the bone fragments, in all patients the surfaces of the nonunions were drilled according to Beck. A retrospective study design was chosen as a result of our intent to relatively quickly make known to many readers significant beneficial effects of osteogenon as an adjunct therapy in the treatment of nonunion with the Ilizarov method, whereas a prospective study would have taken a long time.

Walking with two crutches was initiated on postoperative day one. During treatment, patients gradually increased weight bearing on the treated limb within their pain tolerance levels, until walking with full weight bearing was achieved. Follow-up visits, including follow-up X-rays, were scheduled in 2–6-week intervals. The Ilizarov external fixator was removed once radiological and clinical evidence of bone union was observed at the nonunion site. The radiological criterion of bone union was the presence of at least three of four cortices or trabecular bridging between bone fragments in anteroposterior and lateral views. The clinical criteria were the absence of the following—pain, pathological mobility, and tibial deformity on fixator dynamization and on forcible attempts at movement at the site of nonunion. Once the Ilizarov external fixator was removed, the patients were advised to walk with two elbow crutches, with partial weight bearing on the treated limb, while continuing to wear a brace or a cast for 3–6 weeks to minimize the risk of refracture. Weight bearing was gradually increased, depending on radiological evidence of progress in bone remodeling at the site of nonunion. All patients from both study groups underwent rehabilitation based on the same protocol. Clinical and radiological outcomes were assessed based on medical records compiled during treatment and at a follow-up visit at least 2 years after treatment completion.

### 2.5. Data Collection

In this study, we assessed the following clinical and radiological parameters—duration of Ilizarov fixation, achieved bone union, maintained bone union, time to resuming normal physical activity, time to complete pain relief, the number of patients reporting complete pain relief, the number of patients who were fitted with a cast or splint following Ilizarov fixator removal, and the rate of complications.

The duration of Ilizarov treatment, understood as the time to bone union, was defined as the number of days from Ilizarov fixator mounting to its removal.

Bone union rate was determined on the basis of full available medical and radiological documentation from treatment and follow-up visits; this parameter was expressed as a percentage.

Maintained bone union rate was determined based on available medical and radiological documentation from treatment and the long-time follow-up. This parameter was expressed as a percentage and determined the number of patients in whom bone union was found to be maintained in a long-time follow-up.

Time to resuming normal physical activity was defined as the number of weeks from the day the Ilizarov fixator was mounted to the day the patient returned to work or school.

Time to achieving pain relief was defined as the number of weeks from the day the Ilizarov fixator was mounted to the day the patient discontinued analgesics.

The rate of pain relief—expressed as a percentage—was defined as the proportion of patients who did not take any analgesics over the course of their long-term follow-up.

We assessed the number of patients who were fitted with a cast or splint following Ilizarov fixator removal.

Complication rates were assessed based on the patients’ medical and radiological records. The following complications were taken into consideration—infections, pain, limited range of motion, edema, nerve damage, vascular damage, nonunion, destabilization of the fixator, reoperation, and implant breakage.

### 2.6. Statistical Analysis

Data were statistically analyzed using the Statistica 14.1 package (StatSoft, Poland). The Shapiro–Wilk test was used to check the normality of distribution. The t test for independent samples and the Mann–Whitney U test were used to compare quantitative variables. The chi-square test was used to determine the relationship between qualitative variables. Additionally, effect sizes were reported as mean differences with 95% confidence intervals for parametric tests, Hodges–Lehmann estimated median differences with 95% confidence intervals for non-parametric comparisons, and odds ratios (ORs) with 95% confidence intervals for categorical variables. The level of statistical significance was set at *p* < 0.05.

## 3. Results

There were no differences in sex distribution, age, or nonunion type between the two groups (Table 1). Both study groups (the osteogenon group and control group) were comparable in terms of rates of peripheral artery disease, the proportion of patients with diabetes, the proportion of smokers, and the location and type of nonunion (hypertrophic/atrophic) (Table 1). Neither group included any patients with bone fragment loss, osteoporosis, or limb shortening by >1 cm. In neither of the groups assessed (the osteogenon group and control group), there were no patients with nutritional disorders, and there were no poor or homeless patients.

The mean follow-up period was 34 months (25–44 months). Detailed results of a comparison between the osteogenon group and the control group have been presented in Table 2.

**Figure 2:**
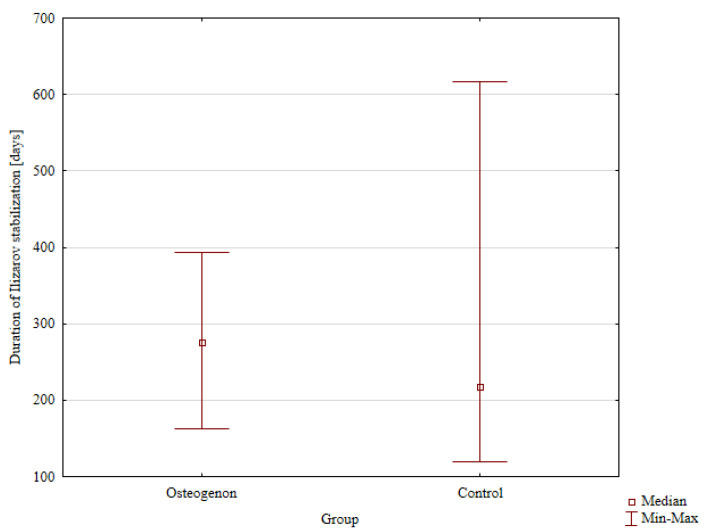
Duration of Ilizarov stabilization in the osteogenon group and control group.

The median time to Ilizarov fixator removal, which corresponds to the time to achieving bone union, was 275 days (162–393 days) in the group receiving osteogenon and 218 days in the control group without osteogenon, with the intergroup difference showing no statistical significance (Figure 2).

**Figure 3:**
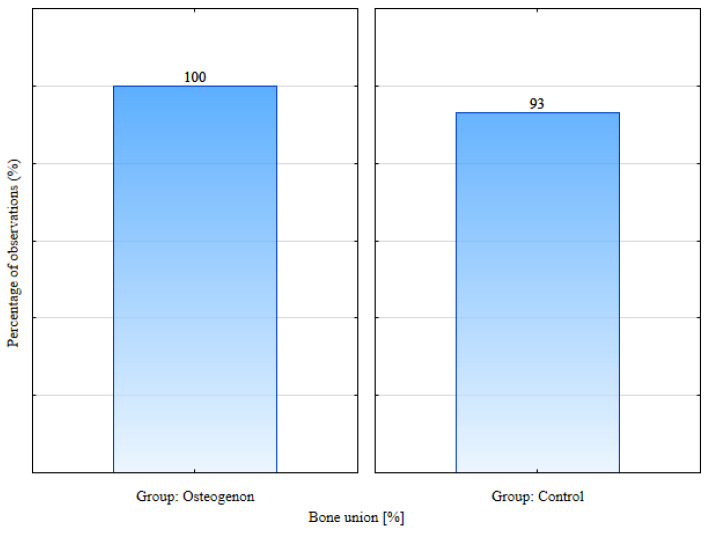
Percentage of bone union in the osteogenon group and control group.

Bone union was achieved in all patients from the osteogenon group and in 93% of patients from the control group. This difference was statistically significant, *p* = 0.041 (Figure 3).

At a follow-up visit at least 2 years after treatment completion, maintained bone union was observed in 85.7% of patients from the osteogenon group and in 79.3% of patients from the control group; however, the difference was not statistically significant.

The median time to resuming normal physical activity was 44.5 weeks in the osteogenon group and 44.5 weeks in the control group. The median time to achieving pain relief in the osteogenon group was lower at 7 weeks than in the control group (12 weeks); however, the difference was not statistically significant. Pain relief was observed in all patients from both groups. Immobilization with a cast or brace after Ilizarov fixator removal was continued in 85.7% of patients from the osteogenon group and in 86.2% of patients from the control group.

A total of 41.9% of patients from the osteogenon group and 44.8% of patients from the control group developed complications. There were 13 complications in the osteogenon group. Six of those cases involved pin-site infections, which required treatment with dressings and oral antibiotic therapy. Four patients required reoperation, which involved Beck’s drilling method and Judet’s osteoperiosteal decortication. Four patients exhibited limited range of motion at the ankle joint, which required intensive rehabilitation and intensive exercises. We did not observe such complications as edema, nerve damage, vascular damage, persistent pain, nonunion, fixator destabilization, or implant breakage in the osteogenon group. There were 13 complications in the control group. Five of those cases involved pin-site infections, which required treatment with dressings and oral antibiotic therapy. Four patients required reoperation, which involved Beck’s drilling method and Judet’s osteoperiosteal decortication. Five patients had limited ankle joint movement, which resolved after intensive exercise and intensive rehabilitation. In the control group, we did not observe such complications as edema, nerve damage, vascular damage, persistent pain, nonunion, fixator destabilization, or implant breakage.

## 4. Discussion

In our study, we assessed the effect of the use of the ossein–hydroxyapatite complex on the treatment of tibial nonunion using the Ilizarov method. In the group of patients using osteogenon, we noted a statistically higher percentage of achieving bone union compared to the control group without osteogenon. The remaining assessed parameters did not differ statistically between the groups. Osteogenon does not significantly affect complication rates, time to fixator removal, time to achieving pain relief, time to resuming normal physical activity, maintained bone union rates, or the proportion of patients who achieve pain relief. The results of our research only partially confirm the research hypothesis.

Tibial nonunion is a common orthopedic phenomenon [1,2,3,4,5,6,7,8,9,10,23,24], which often leads to bone fragment instability and bone and soft tissue loss, consequently associated with pain, difficulty walking, difficulty with everyday activities, lower quality of life, and inability to resume work [1,3,4,8,10,23,24]. Ossein–hydroxyapatite complex has been already reported to have a beneficial effect on bone formation, fracture healing, and osteoporosis treatment [12,13,14,15,16,17,18,19,20,21,22]; however, it may also facilitate fracture nonunion treatment. The organic and inorganic components of osteogenon activate osteoblasts, stimulate bone tissue formation, inhibit osteoclasts, limit bone tissue resorption, accelerate callus formation, and increase bone mass [12,13,14,15,16,17,18,19,20,21,22]. Osteogenon increases serum calcium levels and improves the quality of cortical bone tissue [13,15]. The properties of ossein–hydroxyapatite complex mentioned above may have a beneficial effect on and are expected to improve the effectiveness of nonunion treatment.

Studies analyzing the bioavailability and pharmacokinetic properties of orally administered insulin-like growth factors 1 and 2 demonstrated high serum levels of these compounds maintained for up to 48 h after administration (with the peak concentration reached 4–8 h after administration); this was also observed in patients with renal impairment [25,26,27]. Following oral administration of beta-transforming growth factor, its serum concentration has been shown to remain high for up to 24 h, with the peak concentration reached approximately 3 h after administration [28,29].

There is only one study in the available literature that assessed the use of ossein–hydroxyapatite complex in the treatment of nonunion [15]. Gulnazurova and Kuznetsova, who analyzed treatment outcomes in 15 patients with tibial or femoral fracture nonunion, observed bone union in all treated patients [15]. Time to achieving bone union in the group receiving osteogenon was by 2–3 months shorter than in the control group of 15 patients who did not receive osteogenon [15].

Meleppuram observed bone union in all 42 evaluated patients [4]. Szelerski et al. achieved bone union in all 102 patients treated with the Ilizarov method for tibial nonunion [5]. In their systematic review, Yin et al. reported bone union in 97.5% of patients treated with the Ilizarov method for tibial nonunion [6]. McNally analyzed the outcomes of various surgical techniques used to treat 79 patients with tibial nonunion [7]. The rates of union varied depending on the specific surgical technique and ranged from 73.7% to 96.2%, with the monofocal compression group achieving a refracture rate of 31.6% [7]. Out of 16 patients evaluated by Laursen, 93.75% achieved bone union [8]. Schoenleber reported bone union in all eight patients with tibial nonunion treated with the Ilizarov method [9]. All of the 24 patients assessed by Zhang et al. achieved bone union [10]. Wang reported bone union in all evaluated patients with tibial fracture nonunion treated with the Ilizarov method [11]. Morasiewicz et al. observed bone union in all patients with lower leg bone fractures who received osteogenon and were treated with the Ilizarov method [12]. In our study 100% of patients taking osteogenon achieved bone union, which is a better outcome than those reported in the relevant literature [4,5,6,7,8,9,10,11] and indicates a beneficial effect of ossein–hydroxyapatite complex on fracture nonunion treatment with the Ilizarov method. The rates of bone union in the osteogenon group were higher than in the control group. This may be due to the fact that osteogenon activates bone tissue formation following fractures [15,16] and promotes mitosis of osteocytes in vitro [18].

To date, the only study conducted to assess maintained bone union rates following treatment with the Ilizarov method demonstrated maintained bone union in 95.1% of 102 evaluated patients after a mean follow-up of 7 years [5]. This is a somewhat better outcome than that obtained in our study. The rate of maintained bone union in the osteogenon group at a long-term follow-up was not significantly greater than that in the control group.

Time to Ilizarov fixator removal in 42 patients treated for tibial fracture nonunion was 8–10 months [4]. The mean time to Ilizarov fixator removal in the group of 102 patients assessed by Szelerski et al. was 7.9 months [5]. A systematic review showed a mean time to Ilizarov fixator removal of 9.41 months in patients treated for tibial nonunion [6]. Authors of another study who assessed 79 patients with fracture nonunion treated with the Ilizarov method reported a mean time to fixator removal of 7.5 months [7]. Laursen reported a mean time of Ilizarov treatment of 6 months [8]. Schoenleber observed a mean time to fixator removal of 5.8 months in eight patients with tibial nonunion treated with the Ilizarov method [9]. The authors of another study reported a mean duration of Ilizarov treatment of tibial nonunion ranging from 4.92 to 7.45 months, depending on the study group [10]. The mean time to Ilizarov fixator removal in a group of 15 patients assessed by Wang et al. was 12 months [11]. In another group of 15 patients treated for femoral and tibial nonunion and receiving osteogenon, bone union was achieved 2–3 months earlier than in the group not receiving osteogenon [15]. The osteogenon group in our study was characterized by a median time to fixator removal of 275 days, which is consistent with the data reported in the literature [4,5,6,7,8,9,10,11]. Receiving osteogenon did not affect the time to fixator removal.

Laursen used a brace or a cast in 63% patients following Ilizarov fixator removal [8]. All patients with fracture nonunion treated with the Ilizarov method who were assessed by Schoenleber [9], by Meleppuram [4], and by Wang [11] wore a cast or brace after their Ilizarov fixator was removed. In our study, the proportion of patients who wore a brace or a cast following fixator removal was somewhat lower than those reported in other studies [4,8,9,11]. We did not observe any effect of using osteogenon on the proportion of patients who required a cast or a brace following fixator removal.

Meleppuram reported complications in 90% of the 42 evaluated patients [4]. The complication rate reported by Laursen following treatment of tibial nonunion with the Ilizarov method was 75% [8]. Schoenleber observed complications requiring reoperation in 25% of patients with tibial nonunion treated with the Ilizarov method [9]. The rate of complications observed in another study assessing 24 patients after treatment for tibial nonunion was 20.83% [10]. Wang et al. reported complications in 100% of the evaluated patients [11]. In another study, one-third (33.33%) of patients with tibial or femoral nonunion who were receiving osteogenon developed complications [15]. The complication rates in our study were consistent with those reported by other authors [4,8,9,10,11,15]. The use of osteogenon did not affect complication rates.

In the evaluation and monitoring of the healing processes of fractures and nonunion, inflammatory markers from blood can be used [30]. Moldovan, in his work, evaluated inflammatory markers in patients after stabilization of humeral shaft fractures with open reduction and plate stabilization and with closed reduction and intramedullary nail stabilization [30]. He found that the postoperative aggregate inflammatory systemic index and neutrophil-per-lymphocyte ratio as markers of inflammation are strongly related to the size of surgical trauma sustained during surgery [30]. Similar studies using inflammatory markers in blood could be performed to monitor the healing process of nonunion, especially infected [23,24]. In our study, all patients in both groups had aseptic nonunion.

The median time to pain relief in a group of patients with lower leg fractures treated with the Ilizarov external fixator and receiving osteogenon in a study by Morasiewicz was 21 weeks, which was shorter than in the control group not receiving osteogenon [12]. Patients from our current osteogenon group achieved pain relief sooner (though not significantly sooner) than those in the control group. The shorter (though not significantly shorter) time to achieving pain relief in patients receiving osteogenon than in the control group in our study may be a result of analgesic properties of osteogenon [12,13,14,15]. The analgesic mechanism of action of this drug is not fully understood [12,13,14]. One of the theories draws attention to an association between osteogenon and the process of pain perception, which involves growth factors found in osteogenon [12]. The analgesic effect of osteogenon may be also a result of osteoclast inhibition, since osteoclasts release pain mediators [12].

Meleppuram reported pain relief at a long-term follow-up in 55% of patients with fracture nonunion treated with the Ilizarov external fixator [4]. Laursen observed the absence of pain at a long-term follow-up in 31% of patients with fracture nonunion treated with the Ilizarov external fixator [8]. A total of 88.46% of patients with lower leg bone fractures treated with the Ilizarov method achieved pain relief [12]. Rodianova noted pain relief in 63.3% of patients at 3 months after surgery and in 86.7% of patients at 1 year after surgery, out of 20 patients with fractures treated with external fixation who received osteogenon [13]. Another study showed an absence of pain in a visual analog scale (VAS) 35 days after conservative treatment of distal radius fracture in patients receiving osteogenon [14]. The 15 patients with fracture nonunion treated with the Ilizarov method who were assessed by Wang et al. exhibited a mean VAS pain severity rated at 1.9 at a long-term follow-up [11]. In our study, total pain relief was achieved in all evaluated patients from both groups. The outcomes achieved in our study are better than those reported elsewhere [4,8,12,13,14].

There have been no studies to assess time to resuming normal physical activity after treatment of fracture nonunion. Patients with lower leg bone fractures treated with the Ilizarov method resumed their normal activity after a median time of 22.5 weeks [12]. In our study, patients from the osteogenon group and from the control group resumed their normal activity after a comparable period of time.

Limitations of our study include its retrospective nature, which was due to our incentive to present results quickly, in light of the fact that prospective studies take a long time. Another limitation is the fact that both the osteogenon group and the control group were of small size. This was due to the fact that we wished to assess a homogenous group of patients who were operated on by a single orthopedic surgeon and cases of tibial fracture nonunion are relatively rare. Another limitation of our study is the fact that the patient-reported outcome measurement is not examined. The strengths of our study include a surgical and rehabilitation protocol identical for all patients, all surgeries having been performed by the same orthopedic surgeon, and a long follow-up period. Another strength of our study is the lack of differences between the osteogenon group and the control group in terms of patient age, sex, fracture nonunion types, smoking rates, peripheral vascular disease, diabetes, and nonunion site. In the future, we plan to conduct similar prospective studies with patient-reported outcome measurement, the subjective assessment of patients, also the VAS scale and on a larger group of patients and a larger control group.

## 5. Conclusions

The use of ossein–hydroxyapatite complex has a beneficial effect on fracture nonunion treatment with the Ilizarov method.

The use of osteogenon helps increase the proportion of patients with fracture nonunion who achieve bone union following treatment with the Ilizarov method.

Osteogenon does not significantly affect complication rates, time to fixator removal, time to achieving pain relief, time to resuming normal physical activity, maintained bone union rates, or the proportion of patients who achieve pain relief.

## Figures and Tables

**Table 1:** Detailed characteristics of the osteogenon group and control group.

Analyzed Variable		Osteogenon Group		Control Group		*p* *	RR/OR (95% CI)
		%		%			
Sex	Female	29.03		17.24		0.392	RR = 1.68 (0.53–5.35)
	Male	70.97		82.46			
Type of nonunion	Hypertrophic	92.86		89.66		0.734	RR = 1.04 (0.88–1.23)
	Atrophic	7.14		10.34			
Smokers		38.71		37.93		0.951	OR = 1.03 (0.38–2.75)
Peripheral artery disease		16.13		17.24		0.908	OR = 0.92 (0.26–3.20)
Diabetes		16.13		17.24		0.908	OR = 0.92 (0.26–3.20)
		M	SD	M	SD	*p* **	Mean Difference (95% CI)
Age		47	12.99	48.48	16.35	0.768	–1.48 (–10.34 to 7.38)
BMI		25.38	2.8	25.24	2.44	0.8312	0.14 (–1.25 to 1.53)
Follow-up [months]		34.88	4.82	52.67	28.45	0.09	–17.79 (–38.31 to 2.73)

M—mean; SD—standard deviation; RR—relative risk; OR—odds ratio; CI—confidence interval; *p* *—*p*-value for the chi-square test; *p* **—*p*-value for the *t*-test.

**Table 2:** Selected parameters in the osteogenon group and control group.

Analyzed Variable		Group		Z	*p*		Median Difference (95% CI)
		Osteogenon Group [n = 31]	Control Group [n = 29]				
	Value						
Duration of Ilizarov stabilization [days]	Q1	210	170	1.431	0.152		+57 (–22 to +132)
	Median	275	218				
	Q3	318	288				
Time to return to normal activity [weeks]	Q1	39	36	−0.24	0.152		0 (–6 to +6)
	Median	44.5	44.5				
	Q3	51	57				
Time to achieve pain relief [weeks]	Q1	6	6	−1.036	0.299		–5 (–12 to +3)
	Median	7	12				
	Q3	12	116				
				χ^2^	df	*p*	OR (95% CI)
Pain relief	% of observations	100	100	0	0	1	–(no difference)
Bone union		100	93.1	1.012	1	0.041	OR = ∞ (not estimable)
Union maintained		85.71	79.31	0.255	1	0.613	OR = 1.50 (0.42–5.34)
plaster/brace after removing the Ilizarov fixator		85.71	86.21	0.001	1	0.965	OR = 0.96 (0.25–3.59)
Complications		41.93	44.83	0.148	1	0.902	OR = 0.90 (0.31–2.61)

Z—standardized value of the Mann–Whitney test; χ2—value of the chi-square test statistic; df—degrees of freedom; OR—odds ratio; CI—95% confidence interval; RR—relative risk; Median Difference—estimated median difference (Hodges–Lehmann estimator); *p*-value for the Mann–Whitney U test or chi-square; Q1, Q3—1st and 3rd quartile.

## Data Availability

The data presented in this study are available on request from the corresponding author.
